# Impact of HLA-B*58:01 allele and allopurinol-induced cutaneous adverse drug reactions: evidence from 21 pharmacogenetic studies

**DOI:** 10.18632/oncotarget.13250

**Published:** 2016-11-09

**Authors:** Ran Wu, Yi-ju Cheng, Li-li Zhu, Lei Yu, Xue-ke Zhao, Min Jia, Chang-hui Wen, Xing-zhen Long, Ting Tang, Ai-juan He, Yi-yan Zeng, Zun-feng Ma, Zhi Zheng, Mu-zi Ni, Gong-jing Cai

**Affiliations:** ^1^ Department of Dermatology, The First Affiliated Hospital of Guiyang College of Traditional Chinese Medicine, Guiyang 550000, Guizhou, China; ^2^ Department of Respiratory Medicine, The Affiliated Hospital of Guizhou Medical University, Guiyang 550004, Guizhou, China; ^3^ Department of Respiratory Medicine, The Affiliated Baiyun Hospital of Guizhou Medical University, Guiyang 550014, Guizhou, China; ^4^ Blood Transfusion Department, The Affiliated Baiyun Hospital of Guizhou Medical University, Guiyang 550014, Guizhou, China; ^5^ Prenatal Diagnostic Center, The Affiliated Hospital of Guizhou Medical University, Guiyang 550004, Guizhou, China; ^6^ Department of Infectious Diseases, The Affiliated Hospital of Guizhou Medical University, Guiyang 550004, Guizhou, China

**Keywords:** allopurinol, cutaneous adverse drug reactions, HLA-B*58:01, diagnosis, meta-analysis

## Abstract

Allopurinol is widely used for hyperuricemia and gouty arthritis, but is associated with cutaneous adverse drug reactions (CADRs). Recently, HLA-B*58:01 allele was identified as a strong genetic marker for allopurinol-induced CADRs in Han Chinese. However, the magnitude of association and diagnosis value of HLA-B*58:01 in allopurinol-induced CADRs remain inconclusive. To investigate this inconsistency, we conducted a meta-analysis of 21 pharmacogenetic studies, including 551 patients with allopurinol-induced CADRs, and 2,370 allopurinol-tolerant controls as well as 9,592 healthy volunteers. The summary OR for allopurinol-induced CADRs among HLA-B*58:01 carriers was 82.77 (95% CI: 41.63 – 164.58, P < 10^−5^) and 100.87 (95% CI: 63.91 – 159.21, P < 10^−5^) in matched and population based studies, respectively. Significant results were also observed when stratified by outcomes and ethnicity. Furthermore, the summary estimates for quantitative analysis of HLA-B*58:01 allele carriers in allopurinol-induced CADRs screening were as follows: sensitivity, 0.93 (95% CI: 0.85 – 0.97); specificity, 0.89 (95% CI: 0.87 – 0.91); positive likelihood ratio, 8.24 (95% CI: 6.92 – 9.81); negative likelihood ratio, 0.084 (95% CI: 0.039 – 0.179); and diagnostic odds ratio, 98.59 (95% CI: 43.31 – 224.41). The AUSROC was 0.92 (95% CI: 0.89–0.94), indicating the high diagnostic performance. Our results indicated that allopurinol–SCAR is strongly associated with HLA-B*58:01, and HLA-B*58:01 is a highly specific and effective genetic marker for the detection allopurinol-induced CADRs, especially for Asian descents.

## INTRODUCTION

Allopurinol, a structural analog of hypoxanthine, is an effective xanthine oxidase inhibitor that has been wildly used as antihyperuricemic agent [1]. In general, allopurinol is well tolerated with gastrointestinal discomfort being the most frequent complaint. However, allopurinol causes a variety of cutaneous adverse drug reactions (CADRs) ranging from milder form, such as maculopapular eruption (MPE), to severe cutaneous adverse reactions (SCARs) including drug-induced hypersensitivity syndrome (HSS), Stevens–Johnson syndrome (SJS) and toxic epidermal necrolysis (TEN) [2]. Although SCARs rarely occur, the mortality rate ranges from 5 - 10% in SJS, 10% in HSS, and increases to 30 – 40% in TEN [2–4].

Allopurinol-induced CADRs is regarded as a complex process with interaction between environmental and genetic factors related to drug metabolism and immune responses. Environmental factors such as cigarette smoking, alcohol abuse, drug-drug interactions, pre-existing diseases (e.g., diabetes, chronic kidney disease), and viral infections have been already well studied so far [5]. To investigate the relationship between human leucocyte antigen (HLA) genetic markers and CADRs induced by allopurinol, recent pharmacogenetic studies have shown HLA-B*58:01 allele as the most strong association signal for allopurinol-induced CADRs [6–8]. However, inconsistent findings were subsequently reported [9, 10]. Individual study may have failed to detect difference due to inadequate statistical power, phenotypic heterogeneity, multiple hypothesis testing, and publication bias. Besides, accumulated evidences have been reported in recent years and there is a need to reconcile these data. Furthermore, HLA-B*58:01 genotyping is a cost-prohibitive test for routine clinical practice, which are mainly used in medical research rather than in clinical practice [11]. Moreover, uncertainty still persists about the clinical performance of HLA-B*58:01 genotype for diagnosing of SCARs caused by allopurinol. Here, we conducted a comprehensive meta-analysis from all eligible pharmacogenetic studies to assess the association of HLA-B*58:01 allele in the development of allopurinol-induced CADRs and to evaluate the diagnosis value of CADRs.

## RESULTS

### Literature selection and studies characteristics

The flow of our literature search is shown in Supplementary Figure S1. We identified 308 records after searching different databases. After reviewing the title and abstracts, 287 records were excluded. After full-text review, the remaining 21 studies [7–10, 12–28] were included in our study, with 12,513 individuals in total, including 551 patients with allopurinol-induced CADRs. The 11,962 individuals without allopurinol-induced CADRs were included in these studies as control groups, which comprised 2,370 allopurinol-tolerant controls from 16 matched studies and 9,592 healthy volunteers or general populations from 13 studies. Most studies were conducted among East Asian populations, 2 studies examined individuals of white race [16, 24], and 1 studies evaluated multi-ethnic populations [27]. Ten studies reported the allopurinol dosages data [7, 9, 14, 15, 17, 20–23, 28], while 9 studies [7, 9, 17, 20–23, 25, 28] provided information on allopurinol exposure duration. Most studies (except for the study by Ye et al [14] and study by Zeng [15]) specified the diagnostic criteria for SJS and TEN cases [29, 30]. The main study characteristics were summarized in Supplementary Table S1. Additionally, only the general population data from the study by Hung et al [7] were used in the overall comparison [7] as for sample overlapping.

### Overall association of HLA-B*58:01 with allopurinol-induced CADRs risk

**Figure 1:**
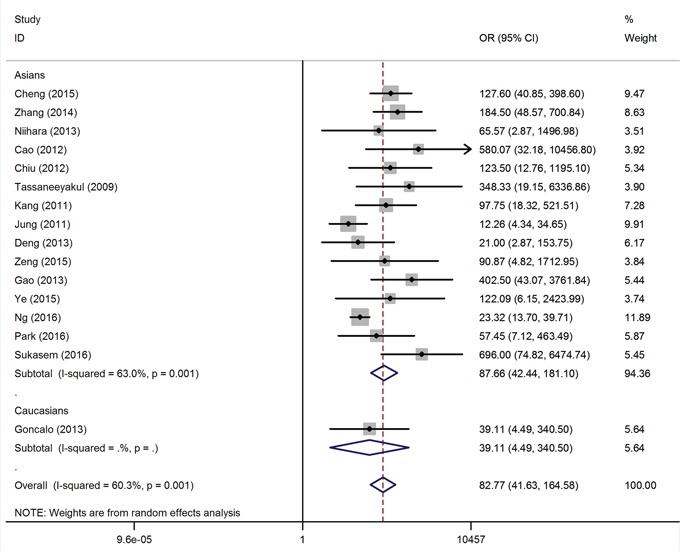
Forest plot for the meta-analysis of the association between HLA-B*58:01 allele carriers and risk of allopurinol-induced CADRs stratified by ethnicity in matched study

**Figure 2:**
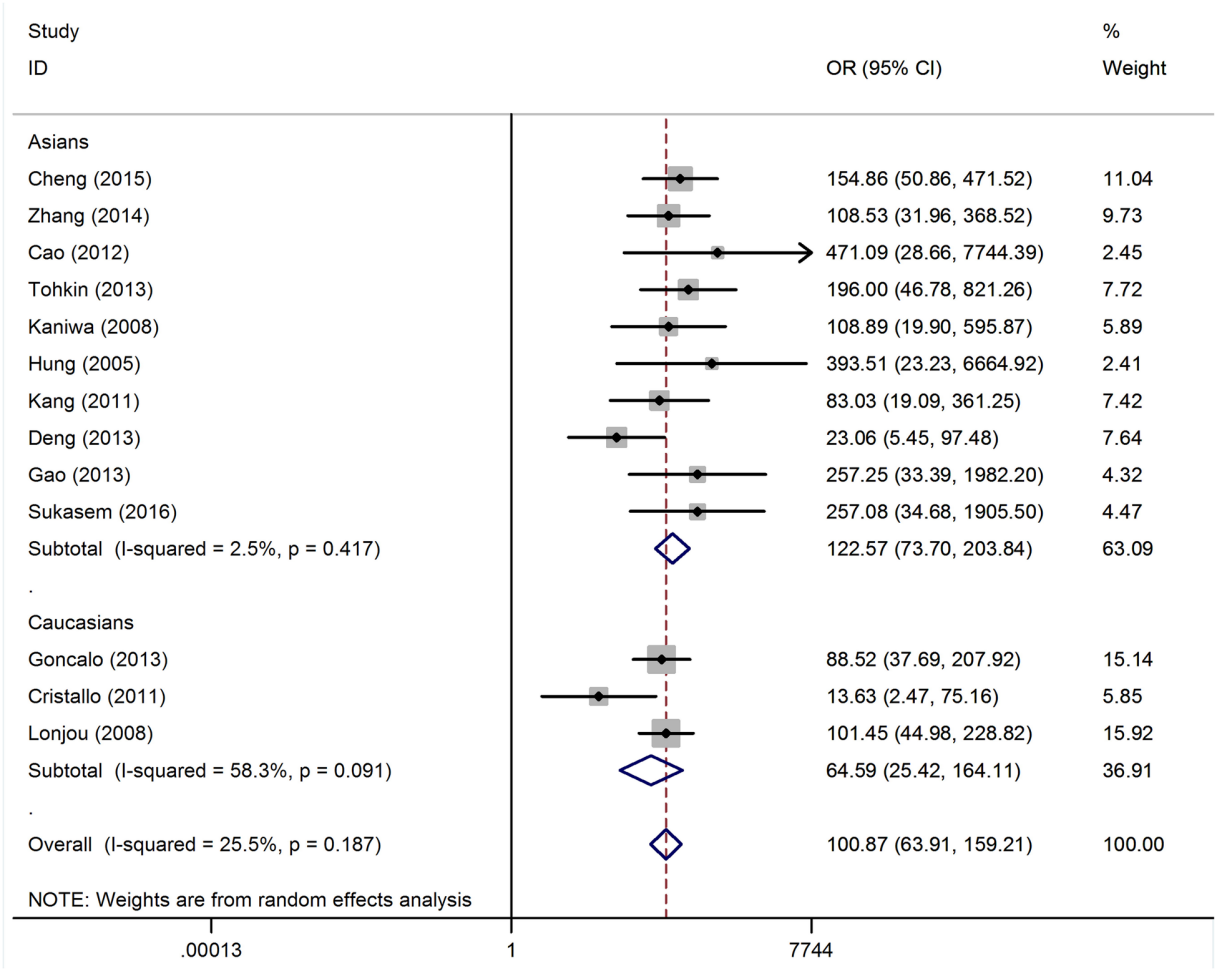
Forest plot for the meta-analysis of the association between HLA-B*58:01 allele carriers and risk of allopurinol-induced CADRs stratified by ethnicity in population based study

Table 1 shows the summary of the meta-analysis for HLA-B*58:01 and allopurinol-induced CADRs. Overall, the HLA-B*58:01 allele showed a strong association with the risk of allopurinol-induced CADRs in matched studies (OR = 82.77, 95% CI: 41.63 – 164.58, P < 10^-5^; Figure 1) and population-based studies (OR = 100.87, 95% CI: 63.91 – 159.21, P < 10^-5^; Figure 2). When only the severe form of CADRs were considered, a significant increased risks of allopurinol-induced SCARs for carrier of the HLA-B*58:01 allele were detected for matched studies and population-based studies with OR of 92.06 (95% CI: 59.54 – 142.32, P < 10^-5^) and 108.39 (95% CI: 73.73 – 159.36, P < 10^-5^), respectively. In addition, significantly increased risk for SJS/TEN was observed among studies using matched control (OR = 79.01, 95% CI: 44.23 – 141.12, P < 10^-5^), and population-control (OR = 106.48, 95% CI: 65.66 – 172.66, P < 10^-5^). When all included studies were stratified based on ethnicity, significantly increased risks of allopurinol-induced CADRs among HLA-B*58:01 carrier were found both in Asians and Caucasians (Table 1). For multiple testing, all associations remain significant after Bonferroni correction. Statistical amount of between-study heterogeneity was found (I^2^ values > 50%); we therefore conducted a meta-regression analysis which showed that the study size may be the source of heterogeneity (P < 0.05). By contrast, source of controls, study quality, age, sex, allopurinol dosage and exposure duration were not correlated with the overall ORs (P > 0.05). To further explore sources of heterogeneity between individual studies, Galbraith plot analyses were used and 2 studies were identified (Supplementary Figure S2).

**Table 1:** Results of meta-analysis for HLA-B*58:01 with allopurinol-induced CADRs

Overall and subgroups analyses	No. of data sets	No. of cases/controls	OR (95% CI)	P(Z)	P(Q)	I^2^ (%)
Study with tolerant controls						
All types of CADRs	16	551/2370	82.77 (41.63-164.58)	<10^-5^	0.001	60.3
CADRs in Asians	15	526/2347	87.66 (42.44-181.10)	<10^-5^	0.001	63.0
CADRs in Caucasians	1	25/23	39.11 (4.49-340.50)	0.001	NA	NA
SCARs	16	466/2370	92.06 (59.54-142.32)	<10^-5^	0.66	0
SCARs in Asians	15	441/2347	95.45 (61.18-148.91)	<10^-5^	0.63	0
SCARs in Caucasians	1	25/23	39.11 (4.49-340.50)	0.001	NA	NA
SJS/TEN	14	211/2207	79.01 (44.23-141.12)	<10^-5^	0.83	0
SJS/TEN in Asians	13	205/2184	81.42 (44.92-147.51)	<10^-5^	0.79	0
SJS/TEN in Caucasians	1	6/23	44.00 (3.18-608.16)	0.005	NA	NA
MPE	6	99/548	29.33 (5.89-145.98)	<10^-4^	0.003	72.1
MPE in Asians	5	93/525	40.45 (6.43-254.57)	<10^-4^	0.002	76.8
MPE in Caucasians	1	6/23	4.40 (0.23-82.98)	0.32	NA	NA
EEM (All Asians)	3	9/112	12.95 (2.30-72.85)	0.004	0.38	0
Study with population controls						
All types of CADRs	13	414/9592	100.87 (63.91-159.21)	<10^-5^	0.19	25.5
CADRs in Asians	10	351/4455	122.57 (73.79-203.84)	<10^-5^	0.42	2.5
CADRs in Caucasians	3	63/5137	64.59 (25.42-164.11)	<10^-5^	0.09	58.3
SCARs	13	381/9592	108.39 (73.73-159.36)	<10^-5^	0.56	0
SCARs in Asians	10	318/3360	147.88 (86.69-252.25)	<10^-5^	0.98	0
SCARs in Caucasians	3	63/5137	64.59 (25.42-164.11)	<10^-5^	0.09	58.3
SJS/TEN	12	190/9312	106.48 (65.66-172.66)	<10^-5^	0.73	0
SJS/TEN in Asians	9	146/4175	156.32 (78.22-312.41)	<10^-5^	0.99	0
SJS/TEN in Caucasians	3	44/5137	58.35 (16.90-201.54)	<10^-5^	0.09	58.7

MPE: Maculopapular eruption; SJS: Stevens–Johnson syndrome; TEN: Toxic epidermal necrolysis.

NA: not available; EEM: erythema exudativum multiforme.

P(Z): Z test used to determine the significance of the overall OR.

P(Q): Cochran's Chi-square Q statistic test used to assess the heterogeneity in subgroups.

MPE and EEM have been considered to be distinct from SJS/TEN, the overall association between HLA-B*58:01 carriers with MPE and EEM is much weaker than SCARs caused by allopurinol. Overall, the random-effect OR of the HLA-B*58:01 for MPE and EEM was 29.33 (95% CI: 5.89 – 145.98, P < 10^-4^; Supplementary Figure S3) and 12.95 (95% CI: 2.30-72.85, P = 0.004; Supplementary Figure S4), respectively.

Under recessive genetic model, we further analysed the gene dosage effect of HLA-B*58:01 on -CADRs induced by allopurinol. The distribution of HLA-B*58:01 genotypes among CADRs cases and tolerant controls were extracted from 4 studies. Patients with homozygous HLA-B*58:01 had 16.78-fold (95% CI: 5.90 – 47.73, P < 10^-5^; Supplementary Figure S5) risk of CADRs compared with individuals with one or no copy of the risk allele.

Sensitivity analyses confirmed the significant association of HLA-B*58:01 with allopurinol-induced CADRs (Supplementary Figure S6). No small study effects were observed according to funnel plot inspection (Egger's test P > 0.05; Supplementary Figure S7).

### Diagnosis value of HLA-B*58:01 on allopurinol-induced CADRs

**Figure 3:**
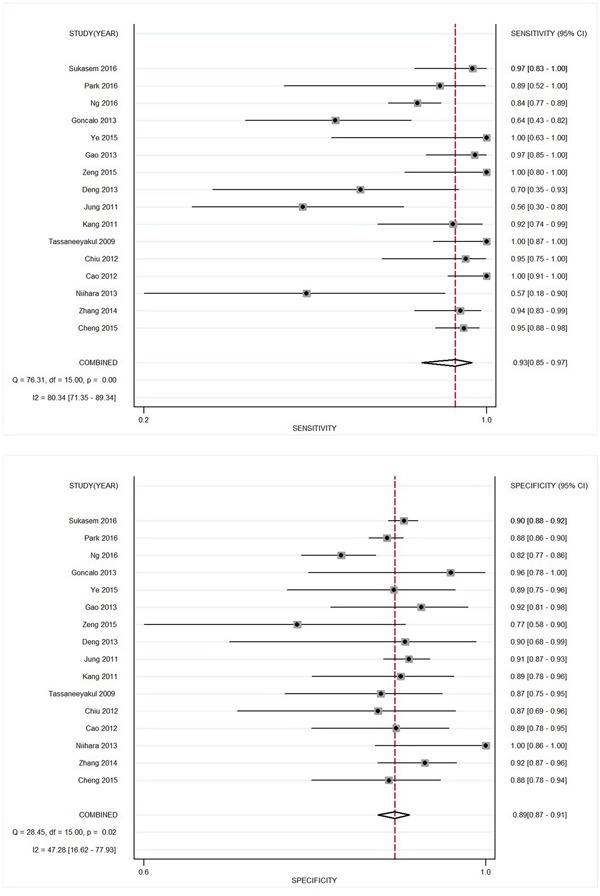
Forest plot of estimates of sensitivity and specificity for HLA-B*58:01 status in the diagnosis of allopurinol-induced CADRs

For diagnosis allopurinol-induced CADRs, the summary specificity was 0.89 (95% CI: 0.87 – 0.91), and the sensitivity was 0.93 (95% CI: 0.85 – 0.97; Figure 3). The pooled PLR was 8.24 (95% CI: 6.92 – 9.81); whereas, the NLR was 0.084 (95% CI: 0.039 – 0.179). The pooled DOR of HLA-B*58:01 was 98.59 (95% CI: 43.31 – 224.41), with significant heterogeneity (I^2^=98.6, Q=21.7, P < 10^-5^). To assess covariates, univariate meta-regression find that ethnic population (P = 0.01) and study size (P = 0.02) may affect the ability of HLA-B*58:01 to diagnosis CADRs.

**Figure 4:**
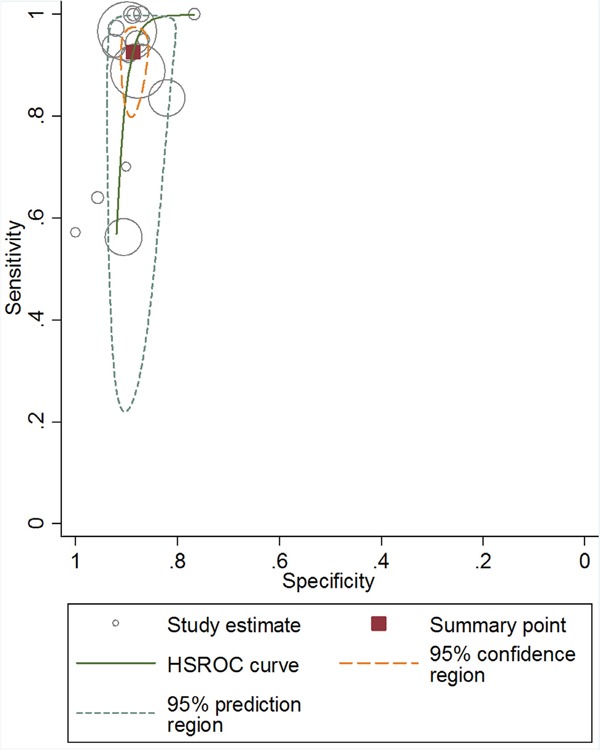
Hierarchical summarized receiver operating characteristic (HSROC) curves of HLA-B*58:01 status for allopurinol-induced CADRs diagnosis

As shown in Figure 4, the HSROC curve indicated a high level of overall accuracy as measured by AUC (0.92, 95% CI: 0.89 – 0.94). The Deek's regression test was performed to detect potential small study effects and no significant selection or publication bias was detected (P = 0.35, Supplementary Figure S8).

## DISCUSSION

Because of the rareness of serious adverse drug reactions, pharmacogenetic studies were underpowered to detect modest differences on small sample sizes. Obtaining adequate numbers of cases with adverse drug reactions for specific drug-gene interaction, and thus improves safety before drug administration is a real challenge. Quantitative synthesis of data from single study, meta-analysis with sufficient power is helpful to address this issue [31]. The present meta-analysis of 21 pharmacogenetic studies, involving 12,513 individuals provided evidence regarding the casual relationship between HLA-B*58:01 carriers and the development of allopurinol-related CADRs; while the association of HLA-B*58:01 allele with with allopurinol-induced MPE or EEM was much weaker.

We observed significant heterogeneity between studies and conducted subgroup analyses and meta-regression to investigate the potential sources. In fact, ethnicity was identified as a potential source of heterogeneity and the effect estimates in Asians (OR range from 73 to 152) was much stronger than that in white populations (OR range from 39 to 64). Thus, differences in genetic background could influence the response to allopurinol. Indeed, the HLA-B*58:01 allelic frequency is much higher in Asians (10 – 15%) than Europeans (1 – 3%) [32]. Due to limited allopurinol-induced CADRs patients investigated in Caucasians, it's possible that different effect estimates among ethnic groups might arise simply by chance as for insufficient statistical power. Therefore, more studies with large sample size are warranted to validate the effect of HLA-B*58:01 on allopurinol-induced CADRs among difference ethnic populations. To check the influence of individual study, sensitivity analysis was performed and showed robust associations even when the largest study was removed.

The area under ROC serves as a global measure of diagnostic performance. According to the suggested guideline for interpretation of area under ROC [33], HLA-B*58:01 had high diagnostic accuracy (0.9 < AUC < 1) for detection of allopurinol-induced CADRs. The DOR combines sensitivity and specificity as one indicator for diagnostic accuracy [34]. In overall analysis, HLA-B*58:01 also showed a high diagnostic performance with a DOR of 90.12. The likelihood ratios are more clinically meaningful indicators [35]. The summary PLR was 8.24 suggesting about 8.3 times higher chance of a allopurinol-induced CADR case to be identified from a positive result for HLA-B*58:01 testing, which was not high enough to be used as a robust diagnostic indicator of allopurinol-induced CADRs. While a negative result for HLA-B*58:01 screening means allopurinol-tolerant control only have 8.6% of probability to develop CADRs. These values indicated that a negative result of HLA-B*58:01 allele could be used as a justification to deny allopurinol-induced CADRs.

As lacks of sufficient evidence about the cost-effectiveness of HLA-B*58:01 typing, and the conflicting results reported, screening HLA-B*58:01 before allopurinol administration is still a remaining issue. Recently, Jung et al. [6] reported that genetic screening test could help to identify HLA–B58:01–positive patients and thus improve safety of allopurinol treatment. More recently, a large cohort study further demonstrated the usefulness of prospective HLA-B*58:01 genotyping for prevention of SCARs induced by allopurinol [36]. Furthermore, two cost-effectiveness studies conducted among Koreans and Thais indicated that HLA-B*58:01 screening before allopurinol treatment is a more cost effective intervention than benzbromarone or febuxostat as an alternative medication [37, 38]. However, Dong et al. reported that allopurinol treatment for chronic gout without HLA-B*58:01 genetic tests remain the optimal strategy from a cost–effectiveness perspective in Singapore [39]. With the development of new technologies and decreasing cost of genetic testing, HLA screening could implement widely and cost effectively in near future.

The detailed pathogenesis mechanisms of CADRs caused by allopurinol remain unknown. Accumulated evidence suggested that T-cell-mediated immunologic response play a central role in CADRs. Through interactions with class I HLA-restricted antigen-presenting cells (APC) and T-cell receptor, CD8-positive cytotoxic T cells believed to trigger an immunologic reaction of SCARs [40]. In vitro study by Lin et al. demonstrates that only APC with HLA-B*58:01 allele present strong cytotoxicity against CD8 + T lymphocytes collected from patients with allopurinol–caused CADRs [41].

Some limitations of the study must be addressed to prevent misinterpretation of our findings. First, substantial heterogeneity was detected and study-level data did not allow us to further explore potential sources of heterogeneity. Second, most cases of allopurinol-induced CADRs were of Asian origin, and results from Caucasian populations may be biased. Third, only single-factor estimates were available and we failed to provide results with further adjustment of potential confounders. For stratified analyses investigating HLA-B*58:01 with MPE and EEM, very few patients were available. Thus, selection bias is inevitable and the results may be easily over inflated. Large studies are needed to address these issues.

Taken together, our meta-analysis from 21 pharmacogenetic studies summarizes the strong correlation of allopurinol-related CADRs with HLA-B*58:01 allele, especially among Asians. Our findings suggested that screening for HLA-B*58:01 may be helpful in allopurinol-induced CADRs detection because of its high level of diagnostic accuracy.

## MATERIALS AND METHODS

### Data sources and search strategy

Pharmacogenetic association studies published before July 2016, on HLA-B*58:01 and CADRs in patients treated with allopurinol were sought by computer-based searches, scanning of the reference lists of all relevant studies and review articles, hand searching of relevant journals. Systematic search of the literature in EMBASE, PubMed, clinicaltrials.gov, The Cochrane Library, Web of Knowledge, MEDLINE, IPA (International Pharmaceutical Abstracts), CINAHL (Cumulative Index to Nursing and Allied Health Literature), and HuGENet (Human Genome Epidemiology Network) used keywords relating to the HLA-B (e.g., “Human leukocyte antigen”) and allopurinol in combination with CADRs (e.g., “drug adverse reaction”, “maculopapular exanthema”, “hypersensitivity syndrome”, “Stevens Johnson syndrome”, “toxic epidermal necrolysis”, and “erythema exudativum multiforme”). No restriction was imposed on the language and the year of publication. Furthermore, citations in the retrieved articles were hand-searched to identify additional relevant reports. The titles and abstracts were read to determine their relevance, and potentially relevant studies were retained for further evaluation. For retrieved articles, the full texts of the articles were read to determine whether they contained information on the topic of interest.

### Selection criteria and quality assessment

Two investigators (R.W and Y.J.C) independently assessed abstracts and titles retrieved from the comprehensive searches for eligible study. Studies included in the current meta-analysis had to meet all the following criteria: (a) original papers containing independent data, (b) investigated the relationship between HLA-B*58:01 and allopurinol-induced CADRs, (c) reported sufficient data for estimating an odds ratio (OR) with 95% confidence interval (95% CI), sensitivity and specificity. Exclusion criteria were as follows: (a) case-only studies, (b) duplicated studies using the same case series, and (c) reviews, editorials, comments, reports from scientific sessions or discussions. A procedure known as ‘Newcastle–Ottawa Scale (NOS)’ has been used to assess the quality of included observational studies. Details are published elsewhere [42].

### Data extraction

Information was carefully extracted from all eligible publications independently by the two reviewers according to a fixed protocol. First author, study design, year of publication, ethnicity, eligibility criteria, diagnosis and phenotypic definition for CADRs patient demographics, CADRs type, dosage of allopurinol and duration of use, genotyping method, HLA-B*58:01 status among cases and controls, results of Hardy-Weinberg equilibrium (HWE) in the control group, and sensitivity and specificity data were collected from eligible studies. Review reports from the two were then compared to identify any inconsistency, and differences were resolved by further discussion among all authors.

### Statistical analysis

The data from each study was divided into two groups according to study design: allopurinol-induced CADRs vs. allopurinol-tolerant patients; allopurinol-induced CADRs vs. healthy controls without allopurinol exposure or subjects obtained from the population database. The strength of the association between the presence of HLA-B*58:01 in at least 1 allele [43] and allopurinol-induced CADRs was estimated using crude odds ratios (ORs), with the corresponding 95% confidence intervals (CIs). The heterogeneity across individual studies was measured by the Cochran's Q test and the inconsistency index (I^2^) [44]. A random-effects model, which is usually more conservative, was used to calculate the pooled ORs [45]. Quantitative assessment of sources of heterogeneity was undertaken by meta-regression analysis using ethnicity, sample size, source of controls, age, sex, allopurinol dosage and exposure duration as covariates [46]. Subgroup analyses by ethnicity (Asians, and Caucasians) and clinical outcomes (SCARs, SJS/TEN, MPE, and EEM) were also performed to seek for potential sources of between-study heterogeneity. Furthermore, the Galbraith plot was used to identify the outliers contributing toward heterogeneity.

Sensitivity, specificity, diagnostic odds ratio (DOR), positive likelihood ratio (PLR), and negative likelihood ratio (NLR) with corresponding 95% confidence intervals (CI) were calculated for each matched study. Meta-analysis of diagnostic test evaluations was performed using standard methods under random-effects model [47]. Hierarchical summary receiver operating characteristic (HSROC) curves were also plotted to graphically present the results [48]. The area under the curve (AUC) results are considered excellent for AUC values of 0.9 – 1.0, good for values of 0.8 – 0.9, fair for values of 0.7 – 0.8, and poor for values of 0.6 – 0.7.

To assess the stability of results, one-way sensitivity analyses were performed by removing each individual study in turn from the total and reanalysing the remainder. Small study effects, such as publication bias, were assessed by inspecting the funnel plots for asymmetry and Egger's linear regression test, as well as Deeks' test [49–51]. Since the P-values of less than 0.05 were considered significant, alpha was firstly set at 0.05, and the Holm-Bonferroni method was used to control the type I error in multiple comparisons with an alpha of 0.0056 (0.05/9). Statistical analyses were carried out using the STATA software version 11.0 (Stata Corporation, College Station, TX, USA).

## SUPPLEMENTARY MATERIALS FIGURES AND TABLE




