# Supplementary material for: Impact of HLA-B*58:01 allele and allopurinol-induced cutaneous adverse drug reactions: evidence from 21 pharmacogenetic studies
Source: Oncotarget. 2016 Nov 9;7(49):81870–9. doi: 10.18632/oncotarget.13250 (PMC5348437; doi:10.18632/oncotarget.13250)
Supplement: Supplementary file 1 [file oncotarget-07-81870-s001.pdf]

Impact of HLA-B*58:01 allele and allopurinol-induced cutaneous adverse drug reactions: evidence from 21 pharmacogenetic studies

SUPPLEMENTARY FIGURES AND TABLE

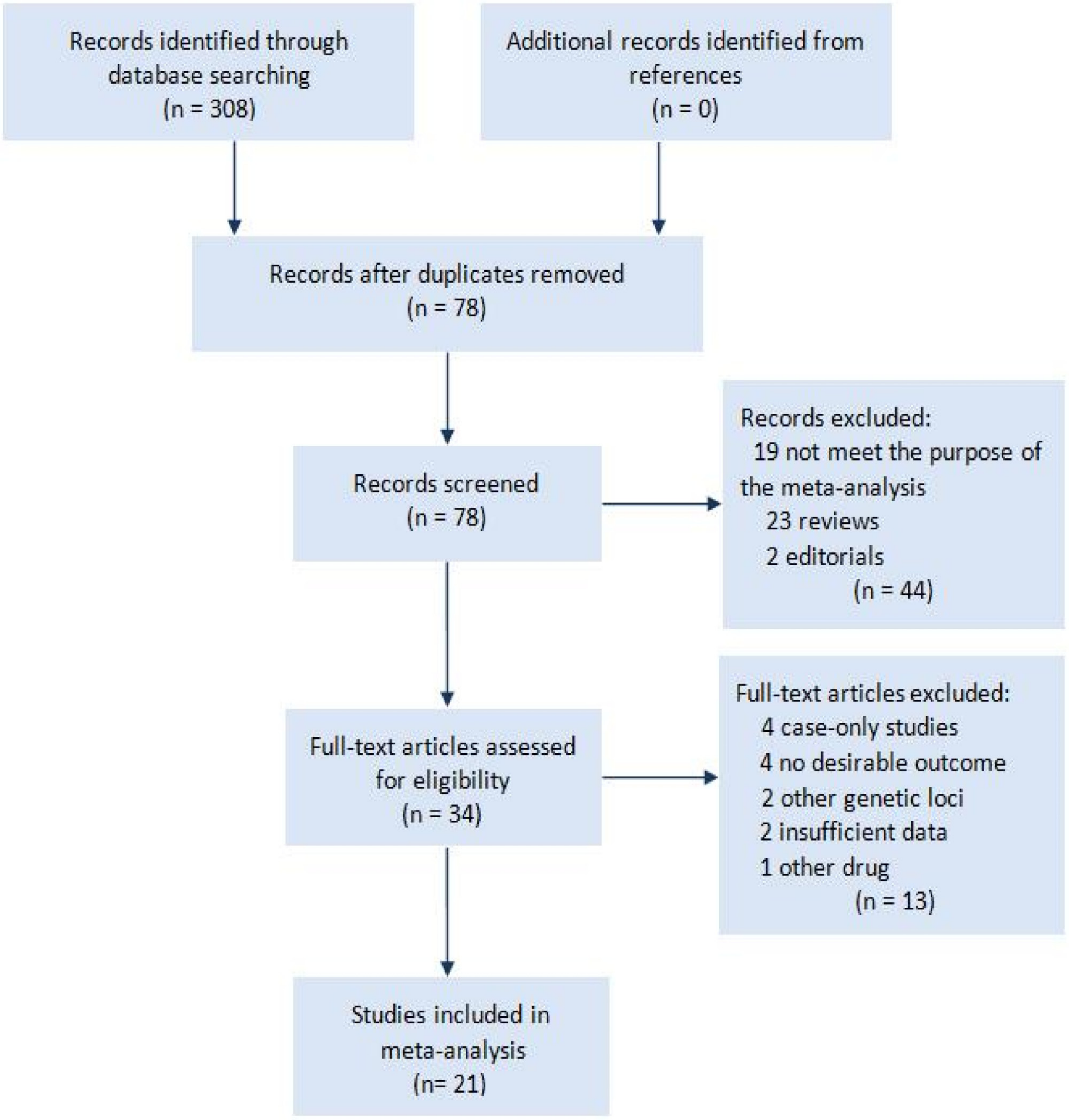

Supplementary Figure S1: Flow chart of literature search for studies examining HLA-B*58:01 and risk of allopurinol-induced cutaneous adverse drug reactions.

(A)

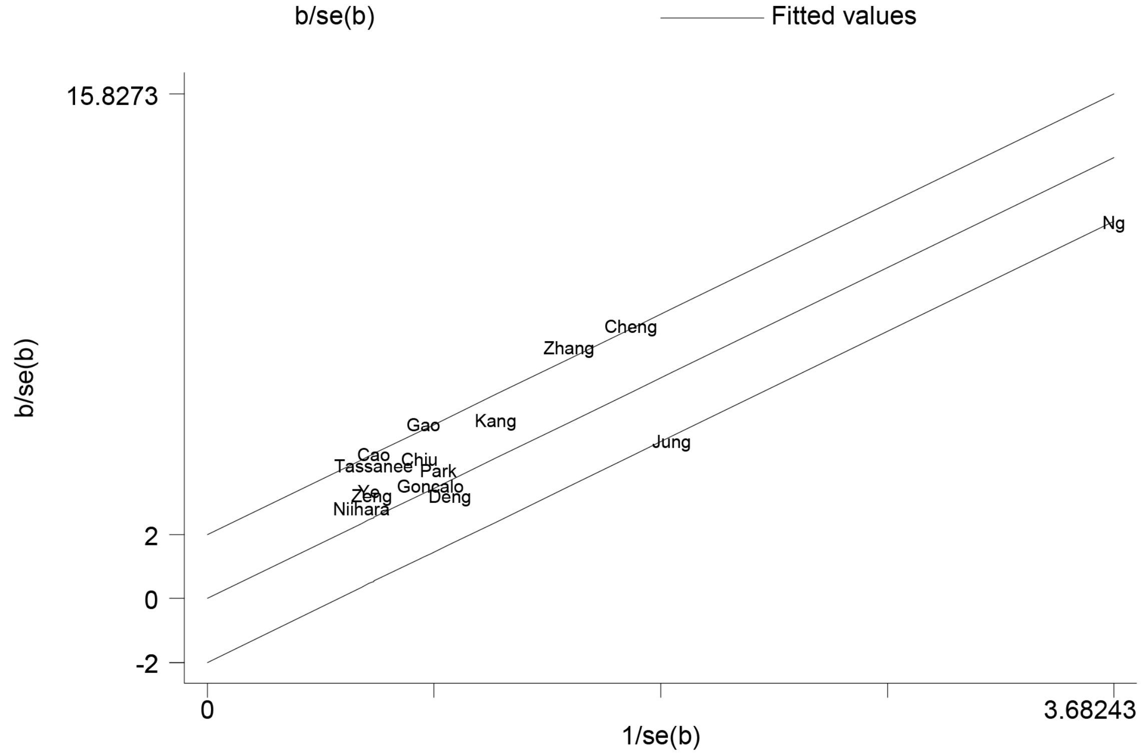

(B)

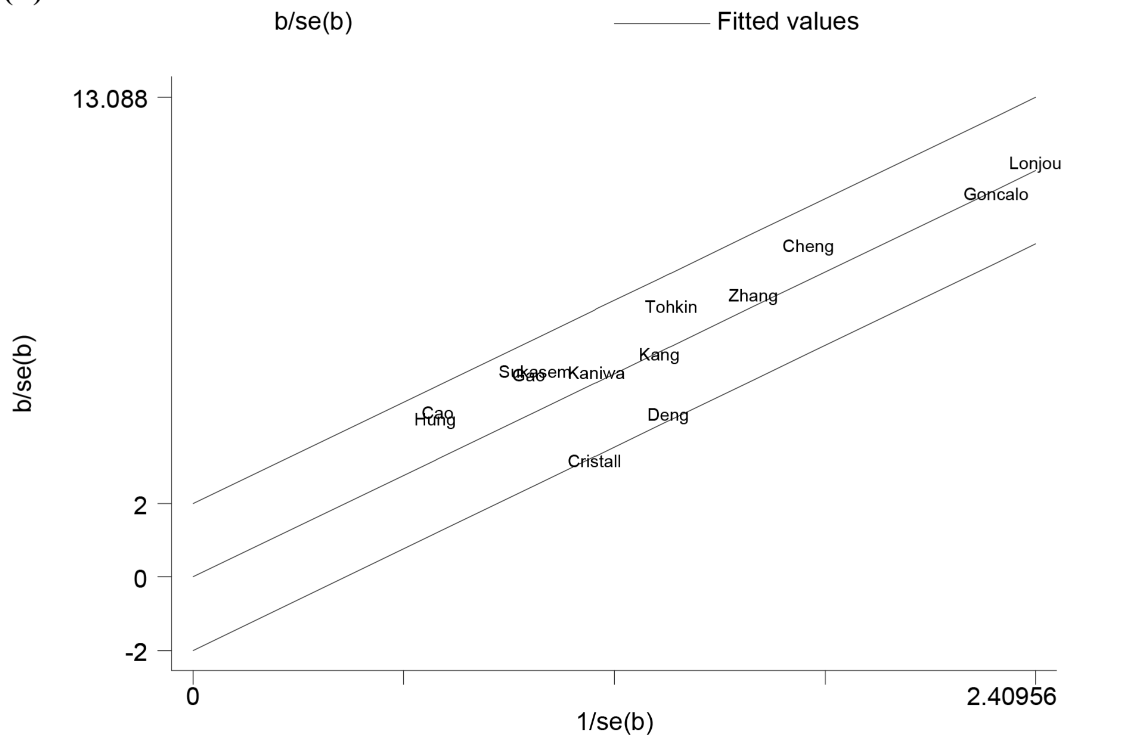

Supplementary Figure S2: Galbraith plots for heterogeneity test of HLA-B*58:01 carriers and risk of allopurinol-induced cutaneous adverse drug reactions for A. matched and **B.** population based study.

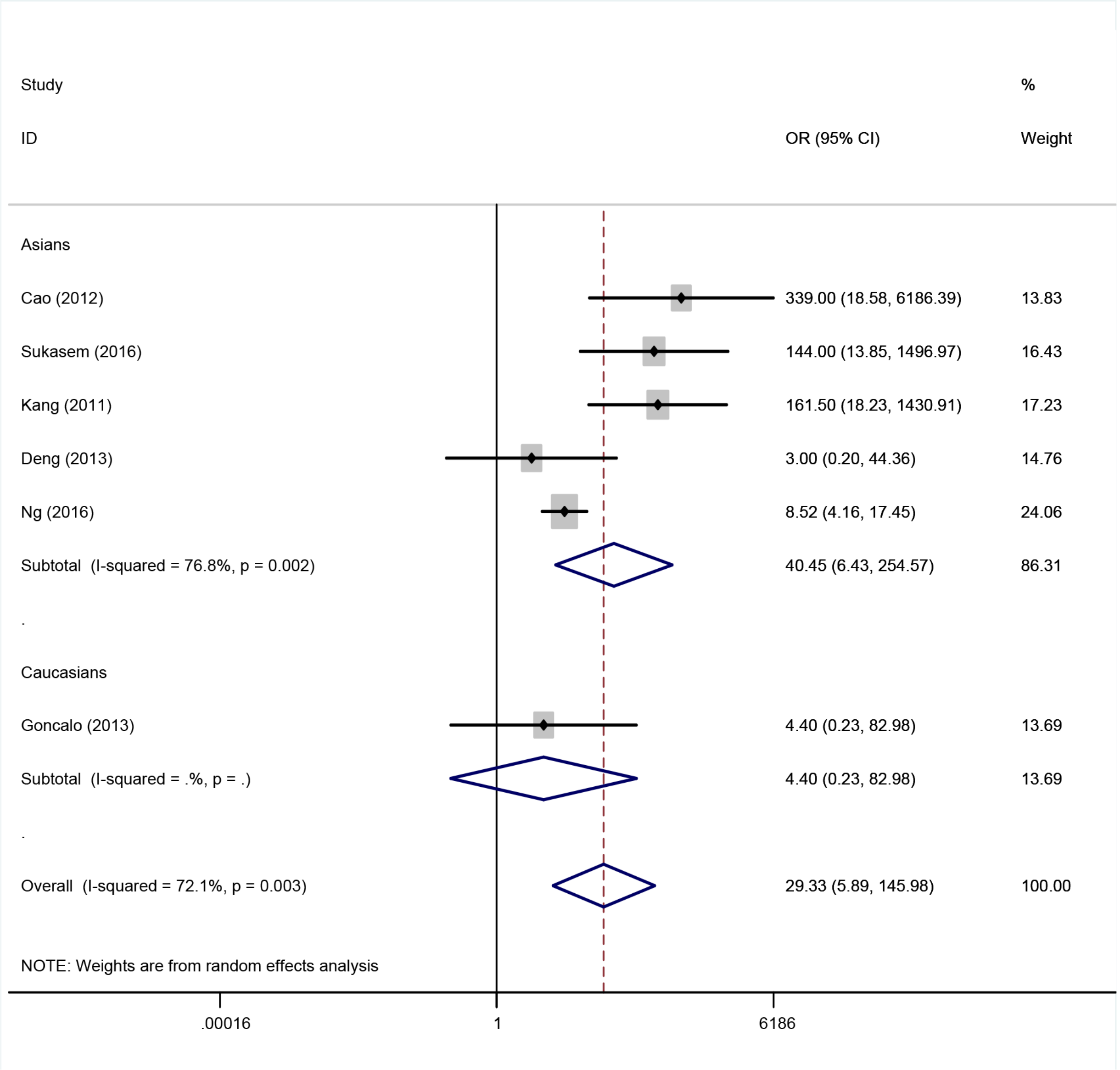

Supplementary Figure S3: Forest plot for the meta-analysis of the association between HLA-B*58:01 allele carriers and risk of allopurinol-induced MPE stratified by ethnicity in matched study.

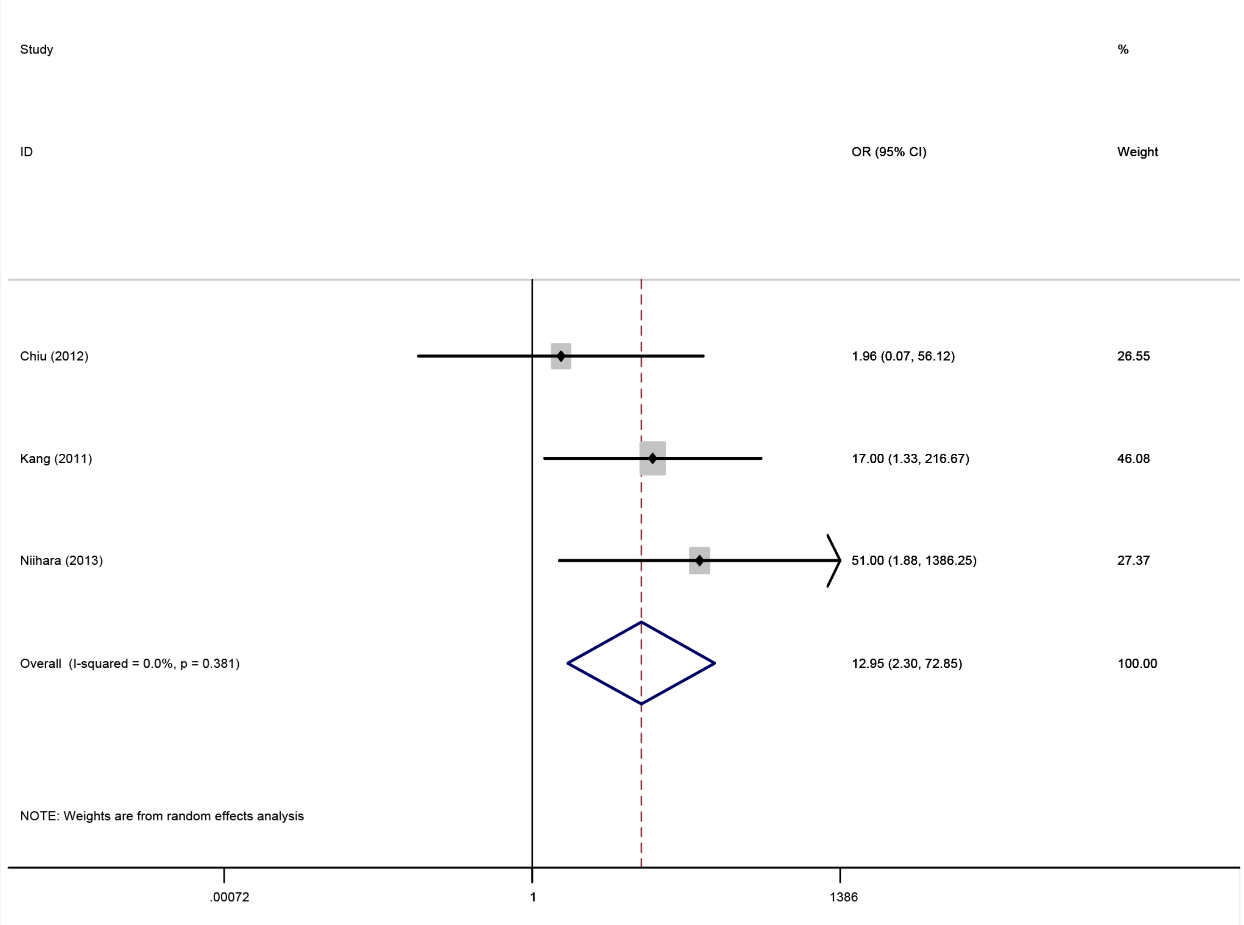

Supplementary Figure S4: Forest plot for the meta-analysis of the association between HLA-B*58:01 allele carriers and risk of allopurinol-induced EEM in matched study.

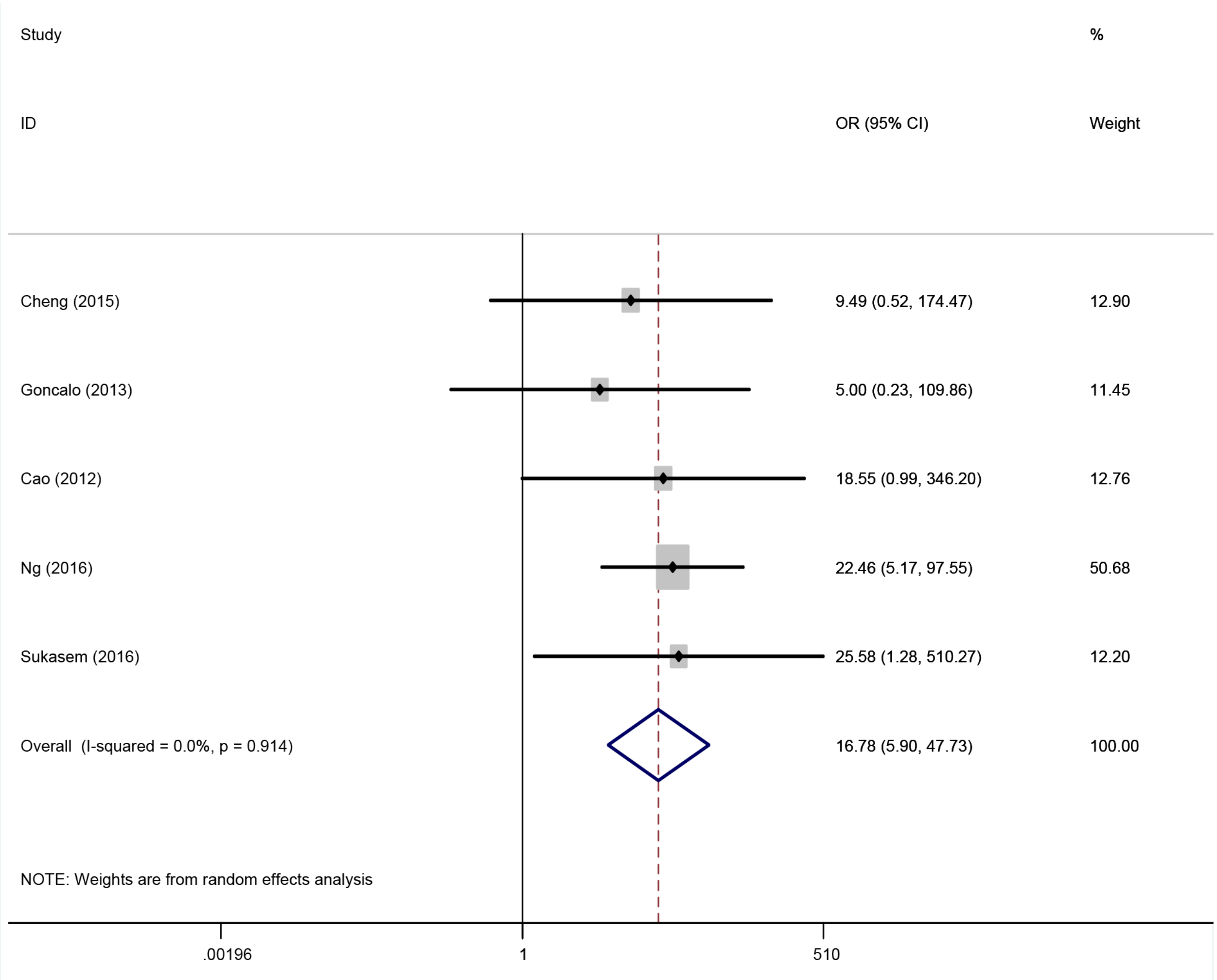

Supplementary Figure S5: Forest plot from the meta-analysis of homozygous HLA-B*58:01 and risk of allopurinol-induced cutaneous adverse drug reactions.

(A)

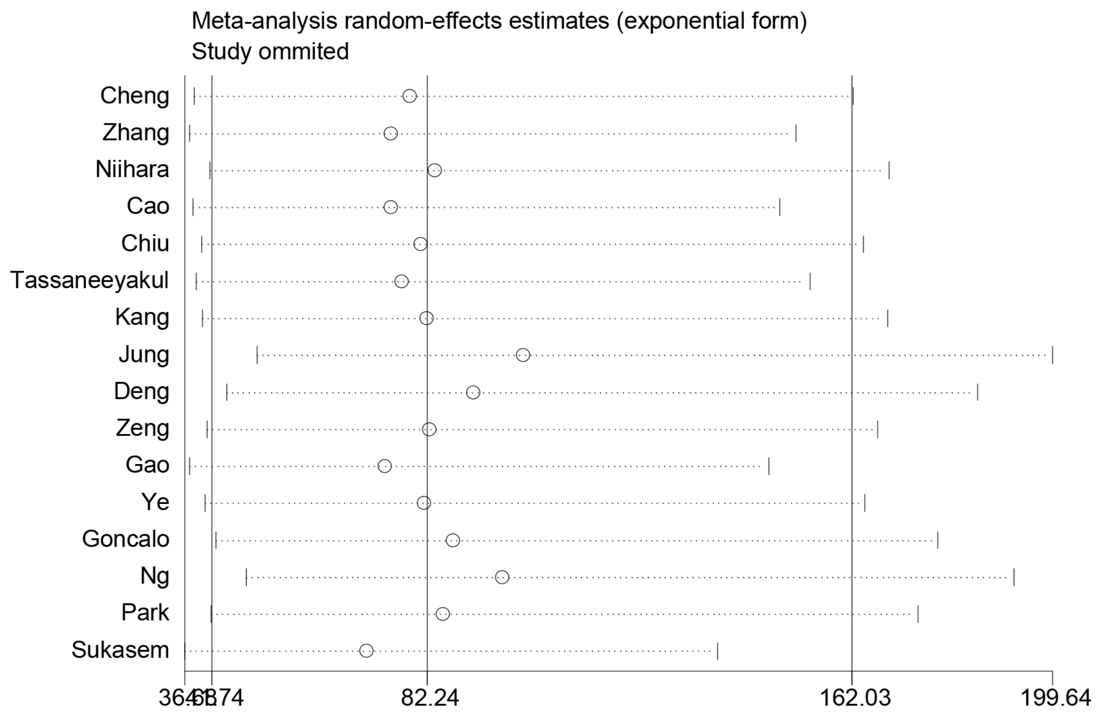

(B)

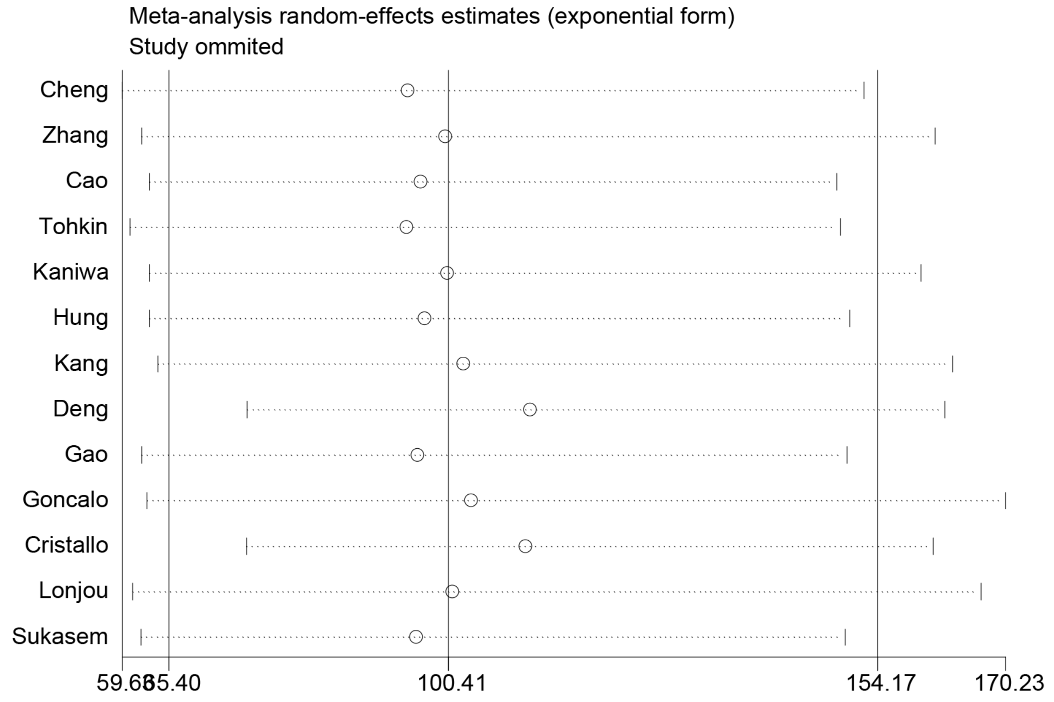

Supplementary Figure S6: Result of sensitivity analyses for HLA-B*58:01 carriers and risk of allopurinol-induced cutaneous adverse drug reactions for A. matched and **B.** population based study.

(A)

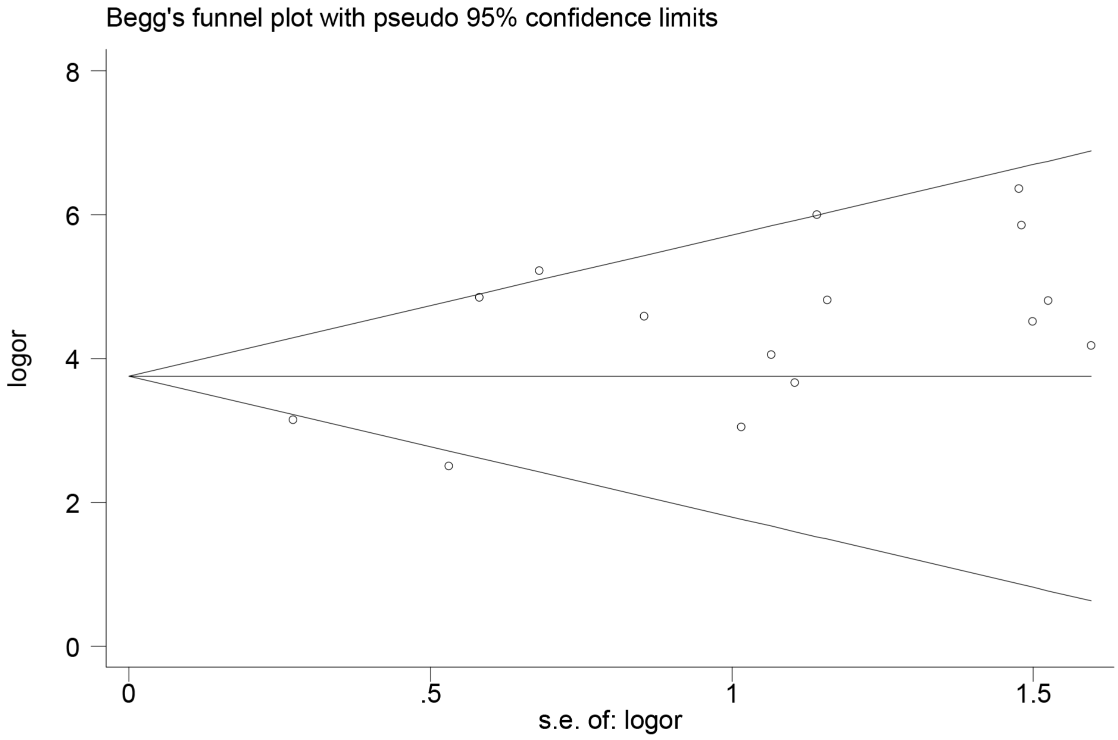

(B)

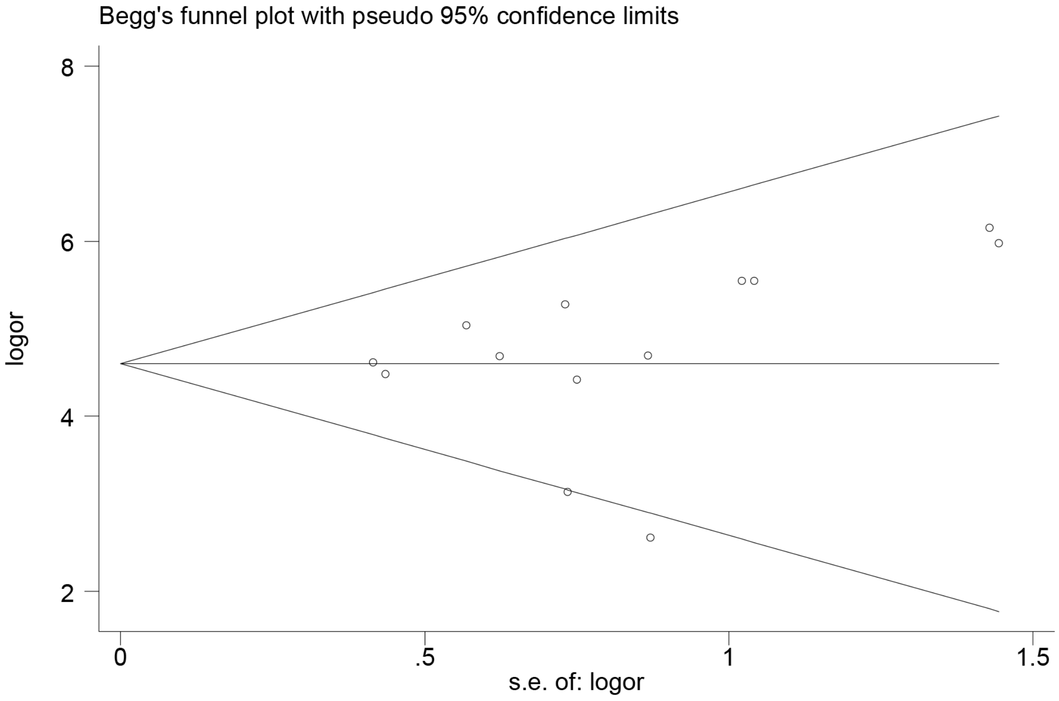

Supplementary Figure S7: Funnel plot of studies of the HLA-B*58:01 carriers and risk of allopurinol-induced cutaneous adverse drug reactions for A. matched (Egger test, P = 0.05) and **B.** population based study (Egger test, P = 0.47).

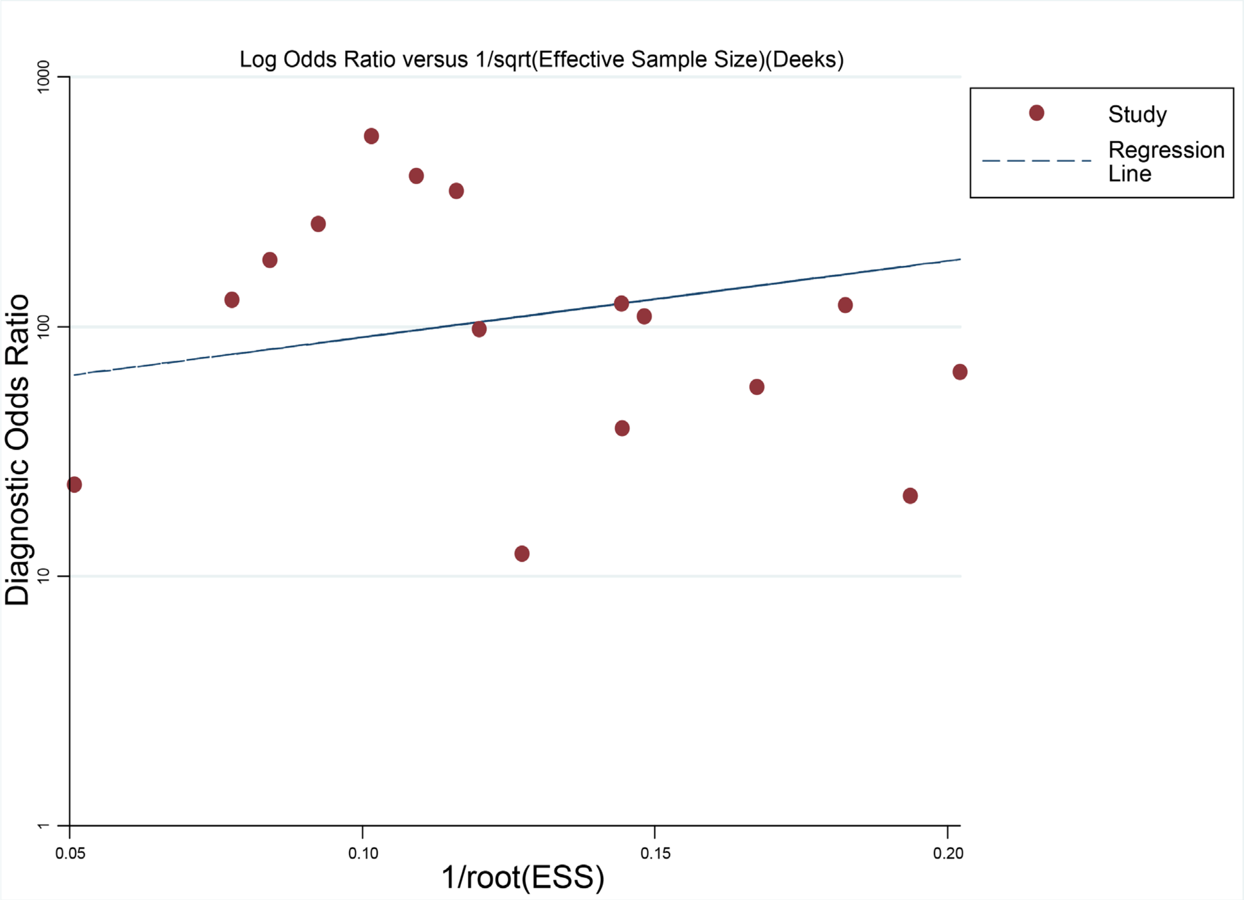

Supplementary Figure S8: The Deek's funnel plot for the assessment of potential publication bias (P = 0.37).

Supplementary Table S1: Characteristics of the studies included in the meta-analysis.

See Supplementary File 1
