# Supplementary material for: Impact of HLA-B*58:01 allele and allopurinol-induced cutaneous adverse drug reactions: evidence from 21 pharmacogenetic studies
Source: Oncotarget. 2016 Nov 9;7(49):81870–9. doi: 10.18632/oncotarget.13250 (PMC5348437; doi:10.18632/oncotarget.13250)
Supplement: Supplementary file 2 [file oncotarget-07-81870-s002.doc]

**Supplementary Table S1** Characteristics of the studies included in the meta-analysis

| Source | Ethnic population | Cases | Controls | Age in cases/tolerant controls | Sex in cases/tolerant controls (%male) | Allopurinol dose in cases/controls(mg/day) | Duration of allopurinol exposure in cases/controls | No. Positive for HLA-B*58:01/Total No. | | | Genotyping method | Quality score |
| --- | --- | --- | --- | --- | --- | --- | --- | --- | --- | --- | --- | --- |
|  |  |  |  |  |  |  |  | Cases | Tolerant controls | Healthy controls |  |  |
| Sukasem, 2016 | Thai | 10 DRESS, 13 SJS-TEN,7 MPE | Tolerant and population | 73/NR | 56.7/NR | 239.3/ NR | 16.4d / >6 m | 29/30 | 4/100 | 111/1095 | PCR-SSOP | 8 |
| Ng, 2016 | Chinese | 46 SJS/TEN; 57 DRESS; 40 MPE; 3 overlapping SJS/TEN and DRESS | Tolerant | 65/64 | 55.0/92.0 | NR | NR/ >6 m | 122/146 | 51/234 | NA | Sequence-specific oligonucleotide reverse line blot | 8 |
| Park, 2016 | Korean | 9 SJS/TEN | Tolerant | 67.7/NR | NR | NR | NR | 8/9 | 116/949 | NA | PCR-SBT | 8 |
| Cheng, 2015 | Chinese | 41 DRESS, 33 SJS, 7 SJS/TEN, 11 TEN | Tolerant and population | 59.1/53.4 | 65.0/95.0 | 186.5/289.8 | 22.0/1153.4 d | 87/92 | 9/75 | 10/99 | PCR-SBT | 7 |
| Ye, 2015 | Chinese | 7 HSS; 1 TEN | Tolerant | NR | NR | 100–200/100–200 | 5–45 d/NR | 8/8 | 5/44 | NA | PCR-SSP | 6 |
| Zeng, 2015 | Chinese | 14 sCADR | Tolerant | 60.1/58.9 | 71.4/80.0 | 100–200/100–200 | NR/ > 14 d | 14/14 | 7/30 | NA | PCR-SSP, PCR-RFLP, PCR-SBT | 6 |
| Zhang, 2015 | Chinese | 48 sCADR | Tolerant and population | NR | NR | NR | NR/ >6 m | 45/48 | 10/133 | 34/280 | TaqMan | 7 |
| Gonçalo, 2013 | Portuguese | 19 DRESS, 2 SJS, 2 SJS/TEN, 2 TEN | Tolerant and population | 67.4/62.0 | 44.0/69.6 | NR | NR | 16/25 | 1/23 | 63/3200 | PCR-SBT | 7 |
| Tohkin, 2013 | Japanese | 7 SJS, 4 TEN | Population | NR | NR | NR | NR | 6/11 | NA | 6/986 | Sanger sequencing | 3 |
| Gao, 2013 | Chinese | 20 HSS, 10 SJS, 3 SJS/TEN, 3 TEN | Tolerant and population | 61.0/74.0 | 61.1/84.0 | 100–300/100–300 | 5–47 d/ >6–240 m | 35/36 | 4/50 | 20/167 | PCR-SSP | 6 |
| Deng, 2013 | Chinese | 6 SJS/TEN, 4 MPE | Tolerant and population | NR | NR | NR | NR | 7/10 | 2/20 | 17/185 | PCR-SSP | 5 |
| Niihara, 2013 | Japanese | 3 SJS, 4 EEM | Tolerant | NR | NR | NR | NR/ > 3 m | 4/7 | 0/25 | NA | PCR-SBT | 5 |
| Chiu, 2012 | Chinese | 6 DRESS, 6 SJS, 7 TEN, 1 EEM | Tolerant | 68.5/71.5 | 55.0/86.7 | 100–600/100–300 | 10–56 d/1–15 y | 19/20 | 4/30 | NA | PCR-SBT | 5 |
| Cao, 2012 | Chinese | 3 DRESS, 8 SJS, 2 SJS/TEN, 3 TEN, 22 MPE | Tolerant and population | 63.8/60.9 | 68.4/90.5 | 100–600/2–60 | 2–60/96–195 d | 38/38 | 7/63 | 80/572 | Sanger sequencing | 6 |
| Kang, 2011 | Korean | 3 SJS, 1 SJS/TEN, 1 TEN, 20 HSS | Tolerant and population | 58.0/51.0 | 56.0/64.9 | 100–600/50–200 | 0.2–5.3/6–72 m | 23/25 | 6/57 | 59/485 | Sanger sequencing | 4 |
| Jung, 2011 | Korean | 2 SJS, 7 HSS, 7 Rash | Tolerant | 41.4/35.9 | 43.8/73.6 | 112.5/100.0 | 59.1/887.1 d | 9/16 | 41/432 | NA | Microlymphocytotoxicity | 6 |
| Cristallo, 2011 | Italian | 3 SJS, 4 TEN | Population | NR | NR | NR | NR | 3/7 | NA | 6/115 | PCR-SSP | 3 |
| Tassaneeyakul, 2009 | Thai | 22 SJS, 1 SJS/TEN, 4 TEN | Tolerant | 65.0/63.5 | 55.6/79.6 | NR | 3–50 d/3–600 m | 27/27 | 7/54 | NA | RT-PCR | 7 |
| Kaniwa, 2008 | Japanese | 7 SJS, 3 TEN | Population | 55/NA | 39.6/NA | NR | NR | 4/10 | NA | 3/493 | Sanger sequencing | 3 |
| Lonjou, 2008 | European, East Asian | 16 SJS, 11 SJS/TEN, 3 TEN | Population | NR | NR | NR | NR | 19/31 | NA | 18/1822 | Sanger sequencing | 3 |
| Hung, 2005^a^ | Chinese | 13 SJS, 5 SJS/TEN, 3 TEN, 30 HSS | Tolerant and population | 66.0/56.0 | 47.1/92.6 | 50–300/100–400 | 1–56 d/6–107 m | 51/51 | 20/135 | 19/93 | Sequence-specific oligonucleotide reverse line blot | 7 |

sCADR: severe cutaneous adverse drug reactions; DRESS: drug reaction with eosinophilia and systemic symptoms; SJS: Stevens–Johnson syndrome; TEN: toxic epidermal necrolysis; HSS: hypersensitivity syndrome; MPE: maculopapular eruption; EEM: erythema exudativum multiforme; NR: not reported; NA: not applicable; d = day(s); m = month(s); y = year(s)
